# Factors influencing medication adherence in multi-ethnic Asian patients with chronic diseases in Singapore: A qualitative study

**DOI:** 10.3389/fphar.2023.1124297

**Published:** 2023-03-09

**Authors:** Sungwon Yoon, Yu Heng Kwan, Wei Liang Yap, Zhui Ying Lim, Jie Kie Phang, Yu Xian Loo, Junjie Aw, Lian Leng Low

**Affiliations:** ^1^ Health Services and Systems Research, Duke-NUS Medical School, Singapore, Singapore; ^2^ Centre for Population Health Research and Implementation, SingHealth Regional Health System, Singapore, Singapore; ^3^ Department of Pharmacy, National University of Singapore, Singapore, Singapore; ^4^ Department of Internal Medicine, Singapore Health Services, Singapore, Singapore; ^5^ Duke-NUS Medical School, Singapore, Singapore; ^6^ Population Health and Integrated Care Office (PHICO), Singapore General Hospital, Singapore, Singapore; ^7^ Post-Acute and Continuing Care, Outram Community Hospital, Singapore, Singapore; ^8^ Department of Family Medicine and Continuing Care, Singapore General Hospital, Singapore, Singapore; ^9^ SingHealth Duke-NUS Family Medicine Academic Clinical Program, Singapore, Singapore

**Keywords:** medication adherence, chronic disease, Asian patients, multi-ethnic setting, WHO framework

## Abstract

**Background:** Poor medication adherence can lead to adverse health outcomes and increased healthcare costs. Although reasons for medication adherence have been widely studied, less is explored about factors affecting medication adherence for patients in non-Western healthcare setting and from Asian cultures. This study aimed to explore cultural perspectives on factors influencing medication adherence among patients with chronic diseases in a multi-ethnic Asian healthcare setting.

**Methods:** We conducted a qualitative study involving in-depth interviews with patients with chronic conditions purposively recruited from a community hospital in Singapore until data saturation was achieved. A total of 25 patients participated in this study. Interviews were transcribed and thematically analyzed. Themes were subsequently mapped into the World Health Organization (WHO) Framework of Medication Adherence.

**Results:** Participants commonly perceived that sides effects *(therapy-related dimension)*, poor understanding of medication *(patient-related dimension)*, limited knowledge of condition *(patient-related dimension)*, forgetfulness *(patient-related dimension)* and language issues within a multi-ethnic healthcare context *(healthcare team and system-related dimension)* as the main factors contributing to medication adherence. Importantly, medication adherence was influenced by cultural beliefs such as the notion of modern medicines as harms and fatalistic orientations towards escalation of doses and polypharmacy *(patient-related dimension)*. Participants made various suggestions to foster adherence, including improved patient-physician communication, enhanced care coordination across providers, use of language familiar to patients, patient education and empowerment on the benefits of medication and medication adjustment.

**Conclusion:** A wide range of factors influenced medication adherence, with therapy- and patient-related dimensions more pronounced compared to other dimensions. Findings demonstrated the importance of cultural beliefs that may influence medication adherence. Future efforts to improve medication adherence should consider a person-centered approach to foster more positive health expectations and self-efficacy on medication adherence, supplemented with routine reviews, development of pictograms and cultural competence training for healthcare professionals.

## 1 Introduction

Medication adherence is defined as “the extent to which a person’s behavior in terms of taking medications corresponds to agreed recommendations from a healthcare provider” (WHO, 2003). Medication adherence can be also viewed as a dynamic process that changes over time and involves three components: initiation, implementation and persistence (Vrijens et al., 2012). Despite the importance of medication adherence, non-adherence to medication remains to be a significant challenge in managing chronic conditions. Studies have shown that up to 50% of patients with chronic diseases fail to take their medications as instructed (WHO, 2003; Kleinsinger, 2018; Foley et al., 2021). In Singapore where this study was conducted, around 60% percent of adults are non-adherent to their chronic disease medication regimen (Lee et al., 2017; Chew et al., 2021). Medication non-adherence creates a considerable economic and clinical burden to individuals and health systems. According to a systematic review by Cutler et al., the annual disease-specific economic cost associated with non-adherence ranged from US$949 to US$44,190 per person (Cutler et al., 2018). In addition, a previous report estimated that poor medication adherence resulted in 200,000 premature deaths in Europe each year (Khan and Socha-Dietrich, 2018). The economic and clinical repercussions of medication non-adherence underscore the importance of developing an optimal mechanism to foster adherence to medication regimen.

Medication adherence is a complex behavior with multiple factors in play. The World Health Organization (WHO) framework for medication adherence categorizes the factors affecting adherence into five major dimensions (WHO, 2003). *Social and economic factors* include the cost of medication and the impact of medicines on patients’ work and social lives. *Healthcare team and system-related factors* deal with healthcare workers’ knowledge, the distribution of medication, and the rapport between doctors and patients. *Condition-related factors* consider the impact of the severity and progression of the illness on medication adherence. *Therapy-related factors* relate to the complexity of the medication regime and possible adverse effects of medication. Finally, *patient-related factors* involve forgetfulness on the part of the patients, perceptions of their conditions, and their attitudes and mindsets, among many things.

The extent of and reasons for medication adherence have been empirically studied in different patient groups using various methods of measurement (Krass et al., 2015; Yap et al., 2016; Durand et al., 2017; Cheen et al., 2019). Literature reported that socioeconomic status and social support were found to be positively associated with medication adherence in adult patients with chronic diseases (Gast and Mathes, 2019; Peh et al., 2021). There have also been several meta-syntheses of qualitative studies exploring patients’ perspectives of perceived barriers to adherence (Marshall et al., 2012; Brundisini et al., 2015; Kvarnström et al., 2021). They highlighted that health beliefs, lay understanding of medication and life context contributed medication adherence. Although these factors are well documented and may apply to Asian patient populations, cultural and healthcare system specificities make it difficult to directly extrapolate from literature. Studies on international variations in medication adherence highlight the need to examine health system context of medication adherence (Rabia Khan, 2018). A recent systematic review also underlines the importance of personal and cultural beliefs that shape the adherence behavior (Shahin et al., 2019; McQuaid and Landier, 2018). However, with a few exceptions (Chen et al., 2009; Abdul Wahab et al., 2021), qualitative studies assessing medication adherence of Asian patients tended to focus on Asian minorities within the Western healthcare context (King-Shier et al., 2017; Jalal et al., 2019; Greenhalgh et al., 2015; Kumar et al., 2016; King-Shier et al., 2018).

Building on existing literature, this study aimed to explore factors influencing medication adherence among patients with chronic diseases in multi-ethnic Asian healthcare setting of Singapore. The healthcare system in Singapore employs a mixed model of public and private funding to ensure the optimum balance between individual responsibility and social protection. Although approximately 80% of the population obtain their healthcare services from the public health system that offers subsidized healthcare services (Khoo et al., 2014), the country’s primary care sector is being dominated by private general practices (Foo et al., 2021). The annual healthcare spending (4.08% of GDP) is lower than countries in developed Western economies such as the US (16%) and the United Kingdom (10%), yet health outcomes of Singapore are largely comparable with these countries (WHO, 2022). Recently, Singapore has announced the HealthierSG initiative in which residents are encouraged to anchor themselves with a primary care doctor for regular check-ins for chronic disease management (Abraham et al., 2022). Like many of its neighboring countries in Asia, Singapore is facing an increasingly aging population (Department of Statistics Singapore, 2021), and medication non-adherence is found to be associated with an increased risk of hospital admission and healthcare expenditure (Ling et al., 2020). Understanding patients’ perspectives will therefore enable an identification of challenges of medication adherence faced by multi-ethnic Asian patients with chronic diseases and inform the intervention to foster medication adherence.

## 2 Methods

### 2.1 Study design and participant recruitment

We conducted a qualitative study involving in-depth interviews. Patients were eligible if they were 21 years and above (a minor is defined as a person who is below 21 years of age in Singapore) and having one or more of the following chronic diseases: diabetes mellitus, heart disease, cancer, osteoarthritis, asthma, COPD, anxiety or depression, stroke, migraine, vision disorders, or adult-onset hearing loss. These diseases were selected based on the top 20 causes of the total burden of disease in Singapore (Ministry of Health Singapore, 2019). Eligible participants were purposively recruited based on gender, years of medication use, chronic condition, language spoken from inpatient wards at a large Community Hospital in Singapore. Eligible patients were identified from the administrative databases by two of the study team members (medical doctors). Potential participants were approached by a research staff who explained the purpose of the study. Patients in community hospitals generally stay for a few weeks for rehabilitation and discharge planning. Inpatient recruitment enabled the study team to facilitate the process from identification of eligible patients to recruitment to interviews.

### 2.2 Data collection

A semi-structured interview guide was developed based on existing literature (Marshall et al., 2012; Krass et al., 2015) with open-ended questions to probe the participants on the important aspects of medication adherence and reasons behind non-adherence (Supplementary Material). Topics included the extent and reasons for non-adherence, goals and motivations for medication adherence and chronic disease management, barriers to and facilitators of medication adherence and suggestions for improving medication adherence. Interviews were conducted in either English or Chinese by a female interviewer (ZL) who had credentials in Bachelor of Science and ample experience in in-depth interviews and focus group discussions in various clinical settings. The interviewer had no prior relationship with the participants. The interviews lasted 35 min–65 min in duration. A couple of participants had caregivers in attendance, but caregivers did not participate in the interviews. We originally planned to conduct face-to-face interviews. However, due to the safe distancing measures enforced during the COVID-19 pandemic, we conducted remote interviews *via* online video conferencing or phone call after the 12th interview. Field notes were taken by the study team member (WY).

### 2.3 Data analysis

All interviews were audio-recorded with the consent of the participants and transcribed verbatim. Transcriptions were not returned to participants due to time constraints. Interviews in Chinese were translated into English and verified for accuracy by the bilingual study team members. Data analysis involved a hybrid approach of data-driven inductive coding (Boyatzis, 1998) and template-based deductive coding (Crabtree and Miller, 1999), supported by iterative study team meetings. Inductive coding was first performed independently by two coders (WY, ZL). The coders first familiarize themselves with the data. The interview data was then coded through open and line-by-line coding by naming or defining concepts through close examination of the data. The data was grouped according to various provisional categories and subcategories of factors influencing medication adherence. For example, provisional categories such as being difficult to reconcile work and medication due to long working hours, caregiving responsibility, and house chores were later merged into a category of competing priorities. Each category was constantly reviewed and refined, with any discrepancies being iteratively discussed and resolved with the third study team member (SY).

The categories and sub-categories identified were then mapped into the WHO Framework of Medication Adherence to explore similarities and differences (WHO, 2003). WHO framework is useful for understanding factors contributing medication adherence in the context of chronic disease management in a holistic manner as it recognizes that issues surrounding medication adherence are not limited to a personal dimension but include a wider environment such as healthcare systems. Hence, this study used the framework as a reference point to explore how inductive results were aligned with different dimensions of adherence and whether there were any categories that did not fit one of the dimensions. The WHO framework provides descriptions of five dimensions of medication adherence which facilitated comparing of inductive coding categories with the framework’s dimensions and subsequently assigning them to these dimensions. Consensus meetings involving all study team members were held in an iterative manner to conduct peer review and validation of the overarching dimensions. The study team employed member checking, detailed audit trail and reflexivity at each step to ensure credibility and improve trustworthiness of the data (Lincoln and Guba, 1985). All data analysis was performed using a data management software, NVivo 12. For rigor and transparency, we anchored our methodology according to the Consolidated Criteria for Reporting Qualitative Research (COREQ) (Supplementary Material) (Tong et al., 2007). Participant feedback was not sought due to difficulty contacting patients after discharge.

### 2.4 Patient and public involvement

Patients or the public were not involved in the design, conduct, reporting or dissemination plans of our research.

### 2.5 Ethics

This study was approved by the SingHealth Centralized Institutional Review Board (Ref: 2020/2200). Written consent, which included publication of anonymized responses from the participants, was obtained prior to each interview.

## 3 Results

### 3.1 Participant characteristics

A total of 25 interviews were conducted. Data saturation was reached with 23 participants with no new themes emerging from subsequent interviews. We undertook two additional interviews to ensure that point of information redundancy was achieved. Of 33 patients purposively approached, eight patients rejected participation for reasons of speech impairment, time constraints and physical discomfort due to chronic conditions. The mean age was 66.4 years and 76% were Chinese. Participants had multiple chronic conditions: the majority of the participants had hypertension (68%), followed by hyperlipidemia (60%), diabetes mellitus (40%) and ischemic heart disease (20%). More than half of the participants (61.3%) had been on medication for more than ten years. (Table 1).

**TABLE 1:** Participant characteristics (*n* = 25).

	N (%)
Age (Mean ± SD)	66.4 ± 11.7
Sex	
Female	10 (40)
Male	15 (60)
Race	
Chinese	19 (76)
Malay	4 (16)
Indian	1 (4)
Other	1 (4)
Education	
Tertiary and above	1 (4)
Secondary	11 (44)
Primary and below	13 (52)
Primary language spoken	
English	16 (64)
Chinese	9 (36)
Years of taking medication	
< 5	5 (20.8)
5–10	5 (20.8)
11–20	7 (28.0)
> 20	8 (33.3)
Condition^a^	
Hypertension	17 (68)
Hyperlipidemia	15 (60)
Ischemic Heart Disease	5 (20)
Diabetes Mellitus	10 (40)
Others	9 (36)

^a^ Participants have multiple conditions and therefore the cumulative percentage exceeds 100%.

### 3.2 Factors influencing medication adherence

The factors influencing medication adherence, grouped according to the five dimensions of the WHO framework, are summarized in Table 2. Below, selected quotes were presented based on the representativeness of the themes identified and their informative nature.

**TABLE 2:** Factors influencing medication adherence according to WHO framework.

Item	Representative quotes
Social and economic dimension	
Competing priorities (Family commitments and busy work schedules)	“Sometimes I miss the medication because of my work. I have long working-hours so when I am really busy, I can’t even manage to go down to do the insulin. And oftentimes, I totally forget the timing. I miss the insulin time.” *(#22, M, 58)*
Financial constraints (Cost of medication)	“Familywise, because I’m from a single parent family so if let’s say I have any flare or relapse right, my mom is the only one who’s supporting the family. And the medications are not cheap. In the long-term I don’t think I can afford it” *(#11, F, 25)*
Social demands	“If I’m out, you know, like social gathering, even after the meal, when you chat with your family or your friends, you will also forget the Metformin.” *(#9, F, 60)*
Healthcare team and system-related dimension	
Long waiting time for refills	“Obtaining medication in the hospital is very difficult for me. I have to wait for 1–2 h. It takes so long to get my medication. It’s not only refills. When I was discharged, I had to wait for 2 h to get medication. My child sometimes gets irritated by having to wait so long.” *(#18, M, 73)*
Lack of communication skills in healthcare professionals (HCPs)	“When we got blood pressure, we have to recognize our medicine. One time I got very high BP. The doctor gave me medicine. He said, ‘take twice a day’. The BP never came down. I went back and he said, ‘take four times a day.’ I kept on taking it four times a day. Went again, he said, ‘now you take six times a day’ When it’s six times a day, I got a shock. I came home and talked to my daughter. My daughter and I went to talk to the doctor asking about doses. He then said ‘it doesn’t matter. this is a very low dose’ By then I was already scared, so I never take the medicine*.*” *(#15, F, 77)*
Inadequate patient-HCPs relationship	“…during my post-operation hospital admission, for a few weeks, the nurse didn’t give me a high blood pill. I asked, ‘where is my high blood pill?’. The nurse said, ‘doctor said no need because your high blood pressure is stable.’ ‘are you sure?’ Then I waited for the doctor to come. He said, ‘don’t worry. We always monitor you.’ But when I ever asked polyclinic doctors, they said, “don’t miss! this is long-term’. I am confused, I want to make sure nothing will happen to me.” *(#12, F, 58)*
Language barrier (Difficulty comprehending language(s) used for dispensing labels and instructions)	“Some of them didn’t help me write in Chinese instead. I can’t recognize the English words. My wife’s literacy is also not as good. My children live by themselves. My second daughter-in-law lives next door. I have to bring the medication over to let her or my grandchild help me. It’s cumbersome. So, I just ask them to help me write how many tablets I should take in the morning and in the evening.” (#5, M, 74)
Condition-related dimension	
Comorbidities (polypharmacy)	“I think due to my age and additional conditions that I was facing then after surgery, because I had a total knee replacement surgery, there were times when the medications had to be titrated again. You know, medications had to be adjusted again to the right dose so that my pressure can be controlled. So that’s the challenge.” *(#10, F, 67)*
Deterioration of conditions
	“Initially, my condition was still good, but the hypoglycaemic episodes started happening in the last couple of years after my health had deteriorated. I think it’s because of the injection I am getting aside from the oral medication. It’s difficult to continue.” *(#16, M, 66)*
Therapy-related dimension	
Adjustment to new medication routine	“It’s just that when I started on this medication, I was not used to it because everything came too sudden, so you had to maintain medications, to make sure that you take the medication regularly so it will not flare up. Because there’s no one there to remind me hey you have to take x and y medications, it was very challenging” *(#11, F, 25)*
Discomfort caused by self-injection (pain, bleeding)	“Maybe because of the pain, because when I self-inject myself, I was told that over long term in the stomach, I have to, I don’t self-inject into the same places, if I keep poking into the same places, the skin might be hardened, then I might experience pain.” *(#02, M, 57)*
Administration requirements (dosing at specific times)	“Oh, actually I’m not very good at managing it, to be very frank. Because you know sometimes you are so eager to eat, you forget the medication especially the one before meal. The only thing about Letrozole is you have to take it exactly 24 h later, Yesterday I actually forgot so I take it like 2 h later. It’s not easy” *(#06, F, 63)*
Adverse effects	“Doctor’s instruction is to drink as much as I can, but I can’t [drink] because it will cause diarrhoea the whole day. This medicine is meant for those who want to cleanse their stomach, I cannot take too much. It’s a kind of laxative.” *(#01, M, 57)*
Patient-related dimension	
Poor knowledge of medication dosage and frequency	“When you take your medication, it is alright to miss one day as long as you don’t miss it every day. You have to take your medication but missing one to two days will be alright.” *(#21, F, 78)*
Forgetfulness	“It’s not that I have never forgotten. I might take them a little late. For example, for a medication that I’m supposed to take at 9, I might sometimes take it only at 10 plus. When I realized that I had not taken my medication, I would quickly take them. I don’t really know whether I took the medications or not. I also don’t know whether I took a medication twice on some occasions.” *(#08, M, 72)*
Personal belief	**“**My children are grown up so, I should say I fulfilled my duties. And life is not very important to me because the basic daily necessity is all the same. With all these chronic diseases and medications, I feel very fed-up of life…medication is a waste of money.” *(#25, M, 70)*
Misconception of medication	**“**It’s only when the hospital changes my medication, I’m not sure whether I should start taking the new medication. Like they would change from this type of medication to another type. I will ask others to help me search online for the medication. If they tell me, it’s the same as my previous medication. I will keep my old medication and continue to finish old ones. I would be alright with starting the new medication later.**”** *(#24, M, 77)*
Poor understanding of one’s chronic condition(s)	“In fact, at one time, after taking the cholesterol medicine for about a year or so, I have ideal readings, very good readings, so I thought then I shall skip it and then 6 months later when I went back to see a doctor, the readings were really bad.” *(#06, F, 63)*
Absence of perceived health benefits of medications	“Maybe just on a very psychological level, why people don’t adhere to taking long term medication regularly, I think it’s the poor outcomes. Sometimes, I’ve been taking this medication for years, and yet my sugar level keeps on going up, the doctor keeps on increasing, so there is this giving up of hope.” *(#06, F, 63)*

#### 3.2.1 Social and economic dimension

Participants mentioned that they had missed their medications due to competing priorities such as busy work schedules and family commitments. Having to attend social gatherings was also cited by participants. These activities served as an inadvertent distraction from a daily medication routine and resulted in failure to take the medication as prescribed. For example, participants described that social gathering could be considered as a barrier to medication non-adherence.

Although financial issues were not mentioned as a factor substantially hindering medication adherence, a minority of participants expressed that paying for regular prescriptions could be a burden for the family in the long term and hence would likely to impede medication adherence. As one participant described:

“The new oral medication for my diabetes was so costly as it was not covered by subsidies. My family is not well-off. So I stopped the medication.” (#4, M, 71)

#### 3.2.2 Healthcare team and system-related dimension

The most common factors under the healthcare team and system-related domain included a perceived lack of communication skills in healthcare professionals (HCPs), long waiting times for refills and inadequate relationship between patients and healthcare professionals. Participants commonly stated having to wait for a long time to refill their medication or limited communication skills of HCPs to communicate with patients to elicit their needs and challenges in managing medications. As one participant highlighted, a lack of coordination across multiple physicians prescribing medications along with suboptimal quality of communication made it difficult to manage medications which could result in poor adherence:

“There are some doctors who will take time with the patient if they see that the previous doctor has done something and if they're not very comfortable with it, they will explain why. If they want to take off a medication or add a new medication, they spend the time to explain. Then there are some that are in a rush. You know they just like to briefly glance at the notes. I've had some doctors like this. Although it's written there that I can’t take a certain medication, they still prescribe that exact same medication.” (#15, F, 77)

Another item emerged from the data was the language-related issue. In the multi-ethnic society of Singapore, the primary working language in the healthcare system is English and thus written information is typically generated in English alone. Many older participants mentioned that they had difficulty understanding the instructions because they were not proficient in the language used in the prescription medication labels.

#### 3.2.3 Condition-related dimension

The main challenges under condition-related dimension were the deterioration of conditions and presence of comorbidities. Participants commonly described the complexity of adherence to multiple medications with their comorbid health conditions. For example, a participant spoke of the medications having to be constantly titrated, which she found it challenging to keep up with the changes.

“It's like, if you started by taking, let's say, a very low dose of amlodipine, only one type of medication to control the blood pressure. But later, more and more medications were added to control the pressure. You know, where the medications need to be adjusted because the body's not controlling the blood pressure as well.” (#14, M, 62)

#### 3.2.4 Therapy-related dimension

The most prominent item mentioned under therapy-related dimension was the presence of adverse effects. Those who experienced adverse effects from medications tended to stop their medications. Participants noted that often, they were not informed about the adverse effects and what they should do to counter the medication’s adverse reactions. A participant described:

“Sometimes when the doctor prescribes me some medication, I don’t think they are suitable for me. Sometimes I get a nosebleed when I take the medication. So, I let my daughter-in-law see the medication and she said I cannot take the medication. I will get a nosebleed if I take them. [Interviewer: Then what did you do?] I stop taking the medication and I don’t get a nosebleed.” (#17, M, 73)

Other participants mentioned that they did not adhere to their medications when they did not see any improvement in their chronic conditions. Participants also experienced difficulties when they first started a new medication or had to take medication at specific times.

#### 3.2.5 Patient-related dimension

Across five WHO dimensions for medication adherence, patient-related dimension appeared to be markedly salient in our data. They included poor knowledge of medication dosage and frequency, forgetfulness, personal perception, misconception of medication, poor understanding of one’s chronic condition(s) and absence of perceived health benefits of medications. For example, lack of perceived improvement in one’s condition represented giving up of hope, resulting in cessation of medication intake.

Some participants expressed a fatalistic outlook by stating that they had achieved everything they needed in life and did not see the need to continue taking medications. Similarly, ‘Karma’ was highlighted by a non-adherent participant who believed that having to manage multiple medications was a punishment for his past wrongdoings.

“The number of medicines keeps increasing. I am living alone, and it becomes difficult to keep track of medicines. I don’t know when this [taking medication] will end, so I kind of give up [taking medication]. In my religion, it is called Karma. I think I am punished for my wrongdoings.” (#03, M, 61)

Others had cultural beliefs about Western medication as ‘harm’, making decisions to stop medications or lowering doses at their own discretion.

“To me, medicine is also a poison. So don’t depend on too much. I believe that medicine is not really good for the health. It’s good for the sickness, but it’s not good for the health.” (#04, M, 71)

### 3.3 Suggestions for improving medication adherence

Participants were asked what could help them to adhere to medication. Most of the suggestions were centered around four dimensions of the WHO framework. A summary of factors influencing medication adherence and suggestions to improve medication adherence based on WHO Framework of Medication Adherence can be found in Supplementary Material. For the *social and economic* dimension, participants would like to receive more subsidies from the government to help cover the cost of medical expenses. Participants also felt that reminders from family and friends and assistance from patient support groups would improve their adherence to medications.

Patient support groups can help improve medication adherence. As a patient and also part of patient support group, I always share (with patients) that it is important to adhere to the timing of the medications. The timing has to be very precise. It is very nerve-wracking. And we should always have our medications with us at all times.” (#12, F, 58)

Under the *healthcare team and system-related* dimension, there was agreement that adequate education by HCPs would be important in improving medication adherence. Other suggestions made by participants included improved patient-doctor relationships and enhanced care coordination across the healthcare systems and providers.

“I feel it is important how doctors explain and educate patients. For example, my doctor one day told me, although I am under control, once I start taking the high blood pill, I must take it for life. I didn’t understand, then the doctor drew a diagram on how medicine works and how skipping medications will affect my kidney long-term. From then, I always take the medicine. So, it’s important how doctors educate patients.” (#25, M, 70)

The use of a language that is familiar with patients would be critical for medication adherence.

“I would need someone who can write in Chinese whenever I collect my medications. Some of them write in English but I cannot understand. I would ask them what the medication is for and request them to write down how many tablets I have to take in the morning and in the evening in Chinese.” (#21, F, 78)

For the *therapy-related* dimension, an adjustment to the medication regimen in accordance with one’s lifestyle was found to be helpful.

My previous medication for hypercholesterolemia was supposed to be taken at night but I kept forgetting. I spoke to my doctor, and he changed the medication to one that can be taken in the morning together with my other medications. In this way, I will never forget the medication. So, it is important to find out how best to adjust (the medication regimen) to improve adherence.” (#06, F, 63)

Under the *patient-related* dimension, participants felt that increasing patient motivation and a good understanding of one’s own conditions could help achieve better medication adherence.

“What is important is to understand your own chronic conditions and your medications. You will also need to be motivated to be healthy. I think the hospital, the doctors and pharmacists can talk to patients who are not so compliant and encourage them to stay healthy. Every time I take medication, I always talk to myself ‘I want to be healthy, so I have to obey. I got to keep reminding me of the medication.” (#14, M, 62)

## 4 Discussion

### 4.1 Principal findings

This study sought to understand the factors contributing to medication adherence among multi-ethnic Asian patients with chronic diseases, using the WHO framework of medical adherence. We found that a broad range of factors influenced medication adherence, with therapy- and patient-related dimensions more pronounced compared to other dimensions. In particular, findings from this study demonstrated the importance of cultural beliefs that may contribute to medication adherence. Participants made several suggestions to improve adherence. They included improved patient-HCP relationships and enhanced care coordination across levels, use of language familiar to patients, patient education and empowerment on the benefit medication and medication adjustment.

### 4.2 Implications for research and practice

In our study, therapy-related dimension was the most prominent. In particular, the fear of adverse effects when taking medication was cited as the main barrier. The adverse effects ranged from gastrointestinal discomfort to joint pain and epistaxis; participants commonly reported that they stopped taking medications to prevent unpleasant or harmful reactions to medications. This may be an important factor to consider in a multi-ethnic society since adverse effects can vary with ethnicity (Hsu et al., 2010). Previous studies have found that adverse effects were one of the important reasons for lower rates of medication adherence (Marshall et al., 2012; Ju et al., 2018). A possible strategy to address non-adherence arising from adverse effects would be a benefit-risk counselling whereby physicians evaluate counseling needs of patients, educate steps needed to minimize risks and support patients in the treatment decision making (Kooy et al., 2014). Past research suggests that patients benefit greatly from routine medication reviews through re-evaluation of the patient’s experience at subsequent patient-physician interactions (Zaugg et al., 2018; Xu et al., 2020; Schindler et al., 2020). In addition, a collaborative effort between physicians and pharmacists was found to help patients cope with adverse effects of their medications (Shim et al., 2018). Further research is needed to determine the value of such programs and the most feasible way to implement them in a multi-ethnic Asian healthcare setting to enhance patient adherence.

Patient-related dimension was equally a leading cause of medication non-adherence in our study. Specifically, patient’s cultural perception regarding medication significantly influenced their decision for non-adherence. A specific belief brought up by participants was that Western medications were detrimental to one’s health in the long-term; for this reason, participants often intentionally self-adjusted medication dose or discontinued medications without consulting their doctors. However, we did not observe cultural beliefs on the superiority of traditional medication over Western medication or concurrent intake of both Western and complementary medications, a common belief and practice found in studies of Asian patients in a Western healthcare setting (Li et al., 2006; Eh et al., 2016; Jamil et al., 2022). Our findings are also in contrast with studies in Western healthcare settings where spirituality and religious beliefs were culturally significant factors that were shown to have a considerable effect on medication adherence (Albargawi et al., 2017; McQuaid and Landier, 2018; Shahin et al., 2019). Our finding, together with results from other studies, underlines the importance of understanding cultural perceptions in medication adherence. HCPs should consider the impact of cultural beliefs on medication adherence in diverse ethnic patients when implementing appropriate education strategies (Saha et al., 2013; Bosworth et al., 2017). Developing a program to train HCPs in cultural competence might serve to further promote communication with diverse patients, engaging them as collaborative partners in their care in a multi-ethnic and multi-cultural healthcare setting. Studies have shown that targeted interventions to address specific concerns of cultural groups can enhance adherence and health outcomes (Zolnierek and Dimatteo, 2009; Zeh et al., 2012). Indeed, effective patient education and empowerment were desired by many of our participants. It is therefore timely to implement ongoing support and culturally sensitive educational programs to achieve better medication adherence.

Another culturally relevant factor under the patient-related dimension was patients’ belief about fatalism in our multi-ethnic Asian setting. We found that for some patients, the escalation of medication doses or having to take multiple medications was viewed as meaningless or losing hope, and consequently, there was a gradual sense of aversion to medication intake. Destiny, that seems to be more acceptable in Asian culture, may account for this non-adherent behavior (Heiniger et al., 2015; Kim and Lwin, 2021). Clinicians and healthcare teams should consider how cultural background can impact on health beliefs about medication to ensure that more sensitive care is provided to diverse ethnic patient groups. We also suggest that patients with fatalistic cultural beliefs may benefit from a person-centered approach to foster more positive health expectations, self-efficacy, and health locus of control (extent of an individual’s perceived control over health outcomes on medication adherence) (Bosworth et al., 2017; Gerland and Prell, 2021).

When comparing our results on healthcare team and system-related dimension to recent reviews which assessed factors associated with medication non-adherence (Konstantinou et al., 2020; Kvarnström et al., 2021), we found that language issue was more prominent in our healthcare settings. Having difficulty in understanding the language used by HCPs or written instructions used for medication labels can result in poor communication and suboptimal clinical outcomes. Prior studies reported that limited language proficiency leads to poor medication adherence in migrant or less acculturated minority patients in a Western setting (Kumar et al., 2016; Jalal et al., 2019), What is different in our study is that the language barriers were commonly reported across the board, indicating that patients particularly of older age experienced language issues regardless of ethnic minority status. Greater effort is therefore required to ensure that a language familiar with the patient is used to deliver messages regarding instruction, a suggestion also made by our participants. A combination of pictograms and bilingual text should also be considered to assist communication of medication use as evidenced by previous intervention literature (Berthenet et al., 2016; Malhotra et al., 2019).

Lastly, under social and economic dimensions, financial constraints were acknowledged by some participants who suggested the extension of government subsidies to offset cost concerns. Yet, it should be noted that the majority of participants did not see financial commitments as a determining factor influencing medication non-adherence. This is in contrast to previous studies where cost of medications was a commonly cited factor affecting medication adherence (Briesacher et al., 2007; Khera et al., 2019). A key reason for this difference may be that many medications for common chronic diseases in Singapore are heavily subsidized in public healthcare institutions through various schemes available to patients (e.g., Medisave, MediShield, MediFund and the Community Healthcare Assistance Scheme) that help ease their financial burden (Ministry of Health Singapore, 2021). However, physicians should be aware that a small number of patients might still face cost-related adherence issues so that prescription planning could be done to minimize the cost barriers.

### 4.3 Strengths and limitations

All participants interviewed in this study had at least one disease that falls under the top 20 causes of the total burden of disease in Singapore. Our participants also represented a broad spectrum of the patient population with chronic diseases in terms of the level of education, number of years of taking medications and language spoken. Hence, by obtaining their perspectives on medication adherence, the results of this study provided a meaningful insight into the complexity of factors influencing medication adherence in multi-ethnic Asian patients with chronic diseases.

This study is not without its limitations. First, as participants were recruited from a single intermediary healthcare institution, their experiences might not be generalizable to the spectrum of healthcare services in Singapore. However, many participants recounted their experiences of public primary care services in relation to the topic of medication adherence and hence their perspectives would be relevant. The format of interviews used in this study was limited to individual interviews due to difficulty in scheduling a group discussion. While the questions asked in individual interviews facilitated in-depth open discussions, focus groups would have allowed for a better exchange of views and experiences amongst participants that could have brought about other unique themes (Kitzinger, 1995). While the WHO framework has the benefits of considering a diverse range of factors related to medication adherence, use of other relevant frameworks could have engendered unique insights on medication adherence (e.g., ABC taxonomy for process-oriented medication adherence behavior) (Vrijens et al., 2012). Therefore, more research should be done using different frameworks to explain complex pattens of medication behavior and associated factors. Lastly, despite our efforts to recruit a balanced sample, there was a limited representation of Indian participants in this study, which might have introduced a selection bias in the findings. Nevertheless, the proportion of ethnicity was similar to the distribution of ethnicity in Singapore although Indian participants were slightly under-represented (Department of Statistics Singapore, 2021).

## 5 Conclusion

This study identified a wide range of factors that contributed to medication adherence. Our findings shed light on the importance of cultural beliefs that may affect medication adherence in a multi-ethnic Asian healthcare setting. The factors identified in this study can enable clinicians to understand multiple underlying determinants that promote or hinder medication adherence and incorporate patient concerns and perspectives into appropriate strategies to enhance adherence. healthcare professionals should pay more attention to addressing language barriers and patient’s cultural beliefs through patient education by language-concordant healthcare professionals, succinct written instructions and involvement of trained medical interpreters. In addition, interventions aimed at improving medication adherence will benefit from considering a person-centered approach to foster positive health expectations, locus of control and self-efficacy, along with routine reviews, development of pictograms and cultural competence training for healthcare professionals.

## Data Availability

The datasets presented in this article are not readily available because of participant confidentiality and participant privacy. Requests to access the datasets should be directed to low.lian.leng@singhealth.com.sg.
