# Supplementary material for: Factors influencing medication adherence in multi-ethnic Asian patients with chronic diseases in Singapore: A qualitative study
Source: Front Pharmacol. 2023 Mar 9;14:1124297. doi: 10.3389/fphar.2023.1124297 (PMC10034334; doi:10.3389/fphar.2023.1124297)
Supplement: Supplementary file 1 [file Table1.DOCX]

# Supplementary file 1

# Interview Guide

# Questions about medication adherence

## What are your current chronic conditions, and could you share your daily treatment/medication routine with us?

[Probes]

- *How many medicines are your currently taking for your condition?*
- *What instructions were you given on how to take the medication?*
- *How do you usually manage your medications?*
- *How does the medication affect you in general?*

1. What is your main goal in the management of your chronic condition? What do you hope to achieve good control of chronic condition?

[Probes]

- *What is your main goal of seeking medication?*

## How would you rate your adherence to the current medication regimen?

[Probes]

## *What are some of the reasons for not being able to comply with the recommended routine?*

## Are there any differences in your daily function should you happen to miss a dose and/or round of treatment?

[Probes]

- *How does this affect the way you deviate from the original treatment plan?*

## What are some of the difficulties and challenges in following a medication regimen as prescribed and managing good control of your chronic condition? [use probes only when needed]

- *Socioeconomic Factors*
- *Patient Related Factors*
- *Therapy Related Factors*
- *Health Systems & Services Related Factors*
- *Condition Related Factors*

# Questions about suggestions and interventions (add-on to adherence)

## Based on what you have described regarding some of the difficulties, how do you think patients like yourself can be best supported to ensure that they adhere to the medication regimen? (Elicit responses based on the factors discussed by the participant)

##

## Would you describe the medication treatment that you are receiving from your healthcare provider sufficient in assisting you to achieve good control of your condition?

[Probes]

- *If yes, how has it helped you? If no, why do you think it has not helped you?*
- *How often do you see your doctor? How will you describe your relationship with your doctor?*
- *How do you pay for your medical expenses? Do you have difficulty affording your medication?*

## Are you aware of some programs that foster medication adherence for patients with chronic conditions? (e.g., medication education by pharmacists/nurses in hospitals or polyclinic, Screen for Life program)

[Probes]

## *If yes, how do you think they can be improved to better assist you to follow a medication regimen?*

- *Where and how do you usually obtain information or support?*
- *What other support do you think you will need to improve adherence to the medication regimen?*

## We would like to develop services and reminders that can enhance medication adherence for patients with chronic conditions. What type of services do you think will help overcome some of the difficulties and challenges mentioned previously? Which ones do you prefer and why?

## [probes]

- *Mobile apps*
- *Alarms*
- *Calendar Reminders*
- *Sensor-enabled medication box (information on adherence pattern will be sent to a monitor in the background)*
- *Digital pill box with alarm (no monitoring in the background)*
- *Home visits to sort out medicines or medication box*
- *Community services to educate on chronic conditions and importance of self-management*
- *Tele consult/video consult pharmacists*

1. Is there anything else that we didn’t talk about today that you think important for us to know?

Thank you for your participation.
