# Supplementary material for: Factors influencing medication adherence in multi-ethnic Asian patients with chronic diseases in Singapore: A qualitative study
Source: Front Pharmacol. 2023 Mar 9;14:1124297. doi: 10.3389/fphar.2023.1124297 (PMC10034334; doi:10.3389/fphar.2023.1124297)

Supplementary file 3: Summary on factors influencing medication adherence and suggestions to improve medication adherence based on WHO Framework of Medication Adherence


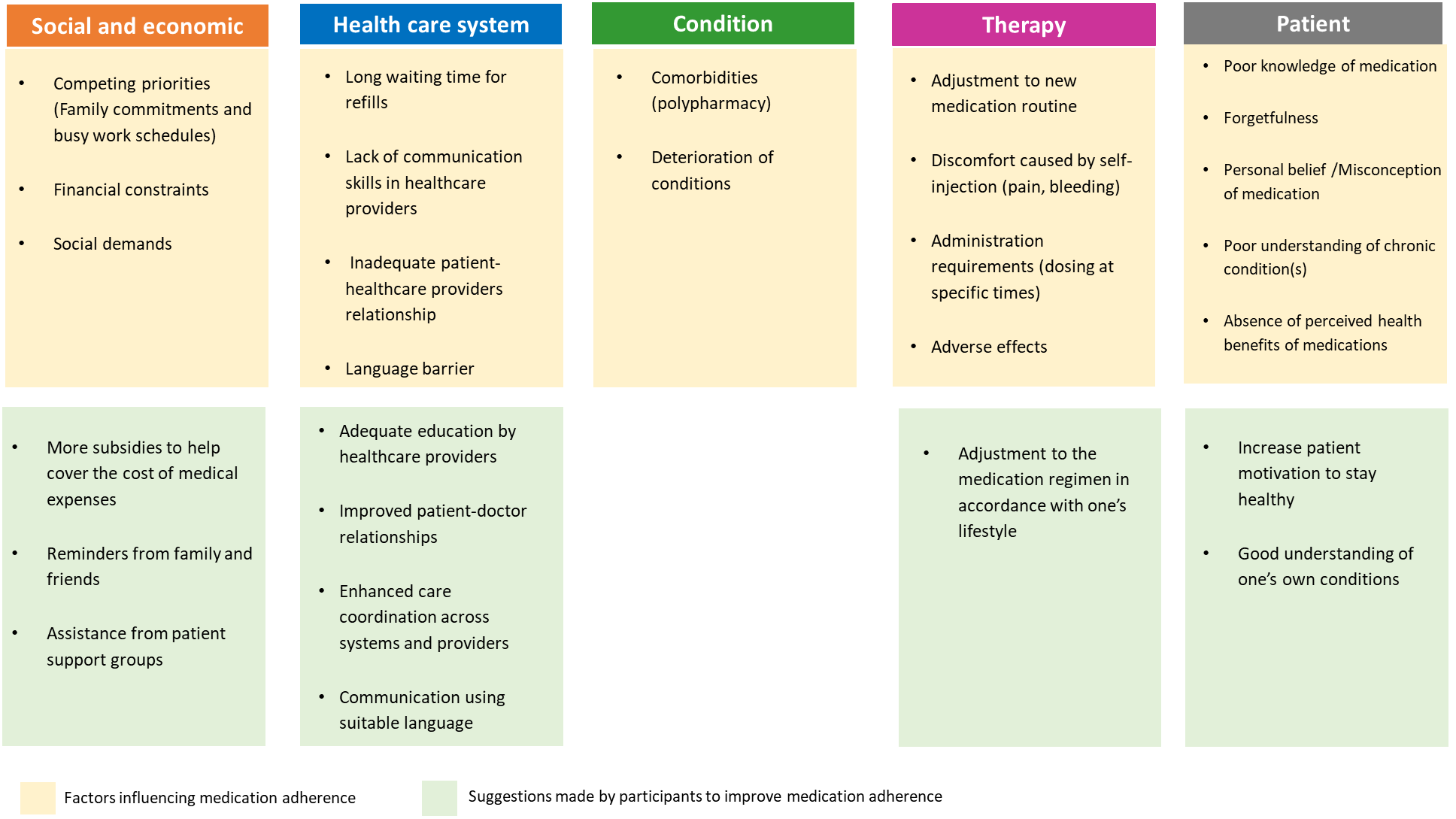

Supplement: Supplementary file 3 [file Table3.DOCX]
